# Popular Errors

**Published:** 1868-09

**Authors:** 


### POPULAR ERRORS.

"To think that the more a man eats the fatter and stronger he will become. To believe that the more hours children study the faster they learn. To conclude that, if exercise is good, the more violent it is the more good is done. To imagine that evey hour taken from sleep is an hour gained. To act on the presumption that the smallest room in the house is large enough to sleep in. To argue that whatever remedy causes one to feel immediately better is good for the system, without regard to more ulterior effects. To eat without an appetite; or to continue to eat after it has been satisfied, merely to gratify the taste. To eat a hearty supper for the pleasure experienced during the brief time it is passing down the throat, at the expense of a whole night of disturbed sleep and a weary waking in the morning."

To dream that "Chambers' Journal," "Home and Abroad," and nineteen hundred and twenty-six other publications can alter the headings of our articles and pass them off as original, or without credit, as is done almost every day, will not be found out, as was the case with the above.

